# A case of basal cell carcinoma arising in the umbilicus^☆^

**DOI:** 10.1016/j.abd.2021.05.023

**Published:** 2022-12-23

**Authors:** Maki Takada, Tatsuhiko Mori, Yuka Hanami, Toshiyuki Yamamoto

**Affiliations:** Department of Dermatology, Fukushima Medical University, Fukushima, Japan

Dear Editor,

Basal cell carcinoma (BCC) is one of the most common cutaneous malignant tumors, and more than half of BCCs arise in sun-exposed areas such as the head and neck region.^1^ Here the authors report a rare case of BCC arising in the umbilicus.

A 72-year-old man was referred to the present study’s hospital, complaining of a pigmented lesion in his umbilicus that appeared one year previously. Physical examination revealed a solitary 15 × 7 mm pigmented nodule (Fig. 1Aa). Dermoscopy showed large blue-gray ovoid nests and the lack of a pigmented network (Fig. 1B). A diagnosis of BCC was made based on biopsy findings. He underwent surgical excision of the tumor with a 3-mm margin under local anesthesia. Both the deep and peripheral margins were tumor-free. Pathological findings of the excised lesions revealed atypical cells that formed nests extending from the epidermis with a palisading pattern. The nests connected to the superficial dermis without apparent invasion, with an artifactual cleft occurring around the nest (Fig. 2). A small number of melanin deposits was observed in the tumor, but not in the stroma. HMB-45, MALT-1, and S-100 were positive in some of the tumor cells. Positive alcian blue and colloidal iron staining revealed mucin deposition in the stroma surrounding the tumor nests. The postoperative course was uneventful, and there was no evidence of recurrence 6 months after surgery.

**Figure 1:**
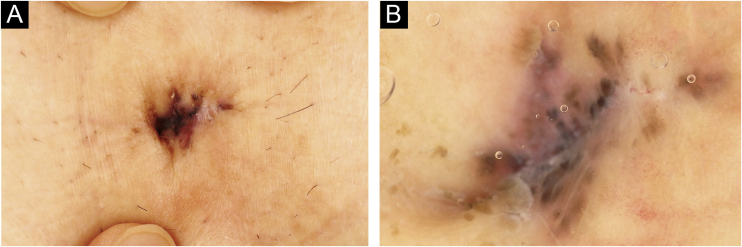
(A) Basal cell carcinoma in the umbilicus appeared clinically as a single black pigmented lesion of 15 × 7 mm in size, with slight scaling. (B) Basal cell carcinoma in the umbilicus showed large blue-gray oval nests with no pigmented network on dermoscopy.

**Figure 2:**
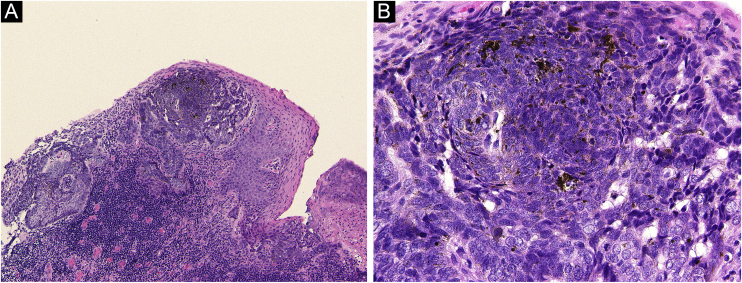
(A) Basal cell carcinoma in the umbilicus was excised, and histopathological examination revealed nests of tumor cells connected to the superficial dermis (Hematoxylin & eosin ×100). (B) Higher magnification shows a small number of melanin deposits in the tumor. (Hematoxylin & eosin, ×400).

Primary umbilical malignancies are rare. Malignant skin tumors affecting the umbilicus include adenocarcinoma of urachal elements, BCC, malignant melanoma, ectopic Paget’s disease, mycosis fungoides, and squamous cell carcinoma.^2^ Previous literature reported that among 112 umbilical tumors, 48 were malignant and 64 were benign.^3^ Among the 48 malignant tumors, 40 were metastases from other organs (the stomach, pancreas, intestines, ovaries, etc.), and 8 were primary malignant tumors (7.1%). Among the 8 primary malignancies, malignant melanoma was the most frequent (4 cases), followed by BCC (2 cases, 1.8%).^3^ Among 518 surgical cases of BCC in the hospital from 2004 to 2020, none involved the umbilicus, and this is the first case. The frequency was estimated to be 0.2% (1/518). According to a survey of BCC in 2010 in Japan,^4^ 1162 of 1578 BCC cases (73.6%) arose in the head and neck region, while 3 (0.2%) arose in the umbilicus. To the best of our knowledge, there are 16 previously reported cases of BCC arising in the umbilicus.^5^ These included 5 men and 11 women, 2.2 times more common in women, and the most common histologic subtype was nodular (9/16), followed by superficial type (3/16). The present case was the superficial type. Pigmented BCC, in which melanin is microscopically detected in the tumor, surrounding stroma, or both, was observed in 2 of the 16 cases. This case clinically presented with a brownish or pigmented nodule, and histopathologically, a small number of melanin deposits was observed in the tumor, but not in the stroma. It has been reported that 48.5% of superficial BCCs occur on the trunk.^1^ Therefore, the superficial type may not be rare in the BCC of the umbilicus. According to a previous report, most patients with BCC had a surgical excision, and the prognosis was generally favorable.^5^ Sun exposure is a risk factor for BCC, but umbilical BCC is not associated with a history of prolonged sun exposure to the area. BCC occurring in the umbilicus is exceedingly rare, but the prognosis after surgery is good. When BCC is suspected, dermoscopy and biopsy should be undertaken.

## Financial support

None declared.

## Authors’ contributions

Maki Takada wrote the initial draft of the manuscript. Toshiyuki Yamamoto assisted in the preparation of the manuscript. Tatsuhiko Mori and Yuka Hanami performed data collection, analysis, and interpretation. All authors have read and approved the final version of the manuscript.

## Conflicts of interest

None declared.
